# A Randomised Trial to Compare the Safety, Tolerability and Efficacy of Three Drug Combinations for Intermittent Preventive Treatment in Children

**DOI:** 10.1371/journal.pone.0011225

**Published:** 2010-06-21

**Authors:** Kalifa Bojang, Francis Akor, Ousman Bittaye, David Conway, Christian Bottomley, Paul Milligan, Brian Greenwood

**Affiliations:** 1 Medical Research Council Laboratories, Banjul, The Gambia; 2 London School of Hygiene and Tropical Medicine, London, United Kingdom; Swiss Tropical Institute, Switzerland

## Abstract

**Background:**

Results from trials of intermittent preventive treatment (IPT) in infants and children have shown that IPT provides significant protection against clinical malaria. Sulfadoxine-pyrimethamine (SP) given alone or in combination with other drugs has been used for most IPT programmes. However, SP resistance is increasing in many parts of Africa. Thus, we have investigated whether SP plus AQ, SP plus piperaquine (PQ) and dihydroartemisinin (DHA) plus PQ might be equally safe and effective when used for IPT in children in an area of seasonal transmission.

**Methods:**

During the 2007 malaria transmission season, 1008 Gambian children were individually randomized to receive SP plus amodiaquine (AQ), SP plus piperaquine (PQ) or dihydroartemisinin (DHA) plus PQ at monthly intervals on three occasions during the peak malaria transmission season. To determine the risk of side effects following drug administration, participants in each treatment group were visited at home three days after the start of each round of drug administration and a side effects questionnaire completed. To help establish whether adverse events were drug related, the same questionnaire was administered to 286 age matched control children recruited from adjacent villages. Morbidity was monitored throughout the malaria transmission season and study children were seen at the end of the malaria transmission season.

**Results:**

All three treatment regimens showed good safety profiles. No severe adverse event related to IPT was reported. The most frequent adverse events reported were coughing, diarrhoea, vomiting, abdominal pain and loss of appetite. Cough was present in 15.2%, 15.4% and 18.7% of study subjects who received SP plus AQ, DHA plus PQ or SP plus PQ respectively, compared to 19.2% in a control group. The incidence of malaria in the DHA plus PQ, SP plus AQ and SP plus PQ groups were 0.10 cases per child year (95% CI: 0.05, 0.22), 0.06 (95% CI: 0.022, 0.16) and 0.06 (95% CI: 0.02, 0.15) respectively. The incidence of malaria in the control group was 0.79 cases per child year (0.58, 1.08).

**Conclusion:**

All the three regimens of IPT in children were safe and highly efficacious

**Trial Registration:**

ClinicalTrials.gov NCT00561899

## Introduction

Although significant advances have been made in controlling malaria during the last two decades, the disease remains a major cause of mortality and morbidity in sub-Saharan Africa. The most important recent steps in improving malaria control include the introduction of artemisinin-based combination therapy (ACT), intermittent preventive treatment (IPT) in pregnancy and increased coverage with ITNs. However, malaria is still estimated to cause directly nearly one million deaths a year and the vast majority of deaths occur among young children, especially among those living in remote rural areas of Africa [1]. The global malaria control strategy includes prompt treatment with effective drugs, effective use of insecticide treated materials, and more recently, a revival in the use of indoor residual spraying (IRS). In addition, the strategy of intermittent preventive treatment (IPT) has become an integral part of malaria control in pregnancy. The IPT principle has subsequently been extended to include infants (IPTi) [2] and children (IPTc) [3]. Recently, IPT in infants was endorsed by WHO as a recommended malaria control strategy in areas with moderate or high transmission and a low prevalence of resistance to sulphadoxine/pyrimethamine (SP).

The role of IPT in the prevention of malaria and anaemia in children has been evaluated in a limited number of trials. In Mali, an area of seasonal malaria transmission, two doses SP given to children aged 6 months to 9 years at an interval of two months gave a protective efficacy of 40% against clinical attacks of malaria [4]. In another study undertaken in Niakhar, Senegal, an area of intense but short seasonal malaria transmission, SP plus one dose of artesunate (AS) given to children less than 5 years old three times at one monthly intervals throughout the peak period of malaria transmission season resulted in an 86% reduction in clinical malaria [3]. Another trial undertaken in Niakhar, Senegal, compared four different treatment regimens. The combinations investigated were SP plus one dose of AS, SP plus three doses of AS, SP plus three doses of amodiaquine (AQ) and three doses of AS plus AQ. The best results were obtained with SP plus three doses of AQ. This regimen showed the highest level of protection against infection and the lowest prevalence of parasitaemia at the end of the malaria transmission season [5]. In another comparative IPTc study undertaken in Ghana, monthly AQ plus AS was compared with bimonthly AQ plus AS and bimonthly SP. Monthly AS plus AQ was the most effective regimen, giving 69% protection against clinical episodes of malaria [6]. These results suggest that IPTc has potential as an affordable malaria and anaemia control tool. SP has been used widely for IPT because of its long half-life, safety, wide availability, low-cost, ease of delivery (a single dose treatment) and a good acceptability profile. However, resistance to SP is increasing, especially in East Africa. . Thus, there is a need to find alternative drugs for IPT.

We have, therefore, undertaken a clinical trial in which the safety, tolerability and efficacy of SP plus AQ, SP plus piperaquine (PQ) and dihydroartemisinin (DHA) plus (PQ) have been compared when used for intermittent preventive treatment in children in an area of seasonal transmission.

## Methods

The protocol for this trial and supporting CONSORT checklist are available as supporting information; see Checklist S1 and Protocol S1.

### Study site and population

The study was based at the MRC Field Station, Basse in the Upper River Region of The Gambia and was conducted between May 2007 and December 2008. Malaria in The Gambia is highly seasonal, occurring almost exclusively during the rainy season (July to November) with greatest incidence in October to November [7]. The entomological inoculation rate is estimated to be in the range of 1 to 50 infective bites per person per year [8]. During the course of the study, first line treatment for uncomplicated malaria in The Gambia was chloroquine plus SP. In 2001, the PCR-corrected treatment failure rate in symptomatic children at day 28 after the start of treatment with SP and chloroquine was 6% in the central part of the country [9]. Since 2008, the first line treatment for uncomplicated malaria has been lumefantrine-artemether (Coartem™, Novartis Pharma, and Basel Switzerland).

### Study Design

The study was designed as an open-label, randomized trial. One thousand and eight children were individually randomized to receive SP plus AQ, SP plus PQ or DHA plus PQ (Duo-Cotecxin®) at monthly intervals on three occasions during the peak malaria transmission season ( September, October, and November). IPTc has been shown to reduce morbidity from malaria in children substantially. Thus, it was considered ethically unacceptable to include a randomized placebo group in the study. For this reason, an alternative design was chosen that relied upon recruitment of children aged 6–59 months from adjacent villages (less than 10 km from a study village). Using the data provided through an on-going Demographic Surveillance System (DSS) 286 children matched for age to children in the trial were randomly selected from 9 adjacent villages to form a control group.

### Screening, enrolment and drug distribution

Meetings were held in the villages in the study area to provide information about the trial to parents of potential study participants and to seek community consent. Fourteen villages agreed to participate in the study; the average distance to the nearest health centre of these villages was approximately 5 km. A list of children living in the study villages and who would be aged between 6 and 59 months at the time of the first treatment in September 2007 was obtained from the Basse DSS. These children and their parents were invited for screening for potential participation in the trial between July and August 2007. Written informed consent was obtained from parents or guardians of all study subjects before screening. Children had their age and identities checked, and were examined to rule out any clinically significant disease. Finger-prick blood was collected for preparation of a thick blood smear and measurement of haemoglobin concentration. Children were considered eligible if they had no clinically significant chronic or acute disease. Exclusion criteria included known allergy to any antimalarial drug or presence of acute or chronic, clinically significant pulmonary, cardiovascular, hepatic or renal disease. Blood films were examined shortly after collection from any child with symptoms suggestive of malaria whilst remaining blood films were examined at a later date. Children with acute malaria were treated with Coartem™ and excluded from the study.

One thousand and eight eligible children whose parents consented were individually randomized into the SP plus AQ, SP plus PQ or DHA plus PQ treatment groups in a 1∶1∶1 fashion. A photo ID card was given to all participants to facilitate identification at every subsequent contact, at home or in the health centres. The randomization list was generated using permuted blocks of 12, using the random number generator of STATA version 10. Treatment assignments were placed in sealed opaque envelopes labelled with the randomization number, each child enrolled was assigned the next study number in sequence.

Monthly treatment was given at the nearest health centre by a team of two nurses who played no part in the evaluation of safety or efficacy measurements and who did not communicate any information on group allocation to the team in charge of the evaluation of adverse events and morbidity. Children received half a tablet of SP [tablets containing 25 mg pyrimethamine and 500 mg sulphadoxine] (Micro Labs Ltd, Hosur, India) if they weighted 10 kg or less and a whole tablet if they weighed more than 10 kg. Amodiaquine [200 mg base tablets] (Micro Labs Ltd, Hosur, India) was given as follows; ½, ½ and ¼ tablets on days 1,2 and 3 for children who weighed 10 kg or less and 1,1 and ½ tablets on days 1,2 and 3 for children who weighed more than 10 kg. Piperaquine [tablets containing 250 mg] (Shanghai Pharmaceutical Co., Shanghai, China) was given at a dose of half a tablet for children who weighed 11 kg or less and as a whole tablet if a child's weight was more than 12 kg. Duo-Cotecxin® [tablets containing 40 mg of DHA and 250 mg PQ] ( Holleykin Pharmaceutical Co. Ltd., Guangzhou, China) was given at a dose of half a tablet for children who weighed 13 kg or less and a whole tablet for children who weighed more than 13 kg.

To ensure compliance with European regulations regarding drug quality, a sample of PQ tablets was tested for drug content and impurities by Sigma Tau Pharmaceutical company; drug concentration was within 5% of the nominal level and analysis of impurities indicated that although some impurities exceeded the ICH limit for known impurities [EMA, 2006], all these impurities were identified and none were of concern in terms of safety. The solubility and drug content of the SP and AQ tablets was confirmed by analysis with high performance liquid chromatography at the London School of Hygiene & Tropical Medicine, which was also used after the trial to confirm that drugs had been allocated to the correct group in accordance with the randomization code. Tablets were crushed, mixed with honey or suspended in water and given on a spoon. All participants were kept under observation for 30 minutes after drugs were taken. If vomiting occurred during this period, the full dose of treatment was re-administrated. If a child vomited the second administration treated was not repeated but he/she was allowed to stay in the trial.

### Surveillance for adverse events

To determine the proportion of children who experienced an adverse event such as headache, fever, weakness, abdominal pain, anorexia, nausea, vomiting, diarrhoea, rash, itching or sleep disorder following drug administration, study participants were visited at home three days after the start of each round of drug administration and a side effects questionnaire completed to document and quantify adverse events that might have occurred since receiving the trial medication. Grading of the severity of adverse events was done by trained field workers. An adverse reaction was graded as mild (grade 1) if it was easily tolerated, moderate (grade 2) if it interfered with normal activity and severe (grade 3) if it prevented normal activity and required treatment.

To help establish whether adverse events were drug related or coincidental, the same questionnaire was administered to children in the control group on completion of each treatment round.

### Morbidity surveillance during the rainy season

Passive surveillance for malaria was maintained throughout the transmission season for 16 weeks (September to December). Parents/guardians of children in the trial and controls were encouraged to take their child to the health centre identified as being closest to their home ( all the health centres in the study area were within walking distance or could be easily reached by local transport) at any time that their child became unwell. Project staff were based at each of these health facilities to identify children in the trial and to ensure that they were seen, properly investigated and treated promptly. At each clinic visit, axillary temperature was recorded using a digital thermometer and haemoglobin concentration measured. A dipstick for diagnosis of malaria (CORE Diagnostics, Birmingham, UK) was used if fever (axillary temperature of ≥37.5°C) or a history of fever within the previous 48 hours was present. In such cases, a thick blood smear was also collected for subsequent confirmation of the diagnosis. Study subjects with documented fever or history of recent fever and malaria parasitaemia were treated with Coartem™. The treatment of study subjects seen at the health centres for other conditions was carried out in accordance with national guidelines and a standardized form was used to document details of the illness and treatment received.

Children enrolled in the study were seen at a health centre at the end of malaria transmission season for examination by a study physician and a finger-prick blood sample was obtained for preparation of a thick blood smear and determination of haemoglobin concentration. A standardized questionnaire was administered to the parents/guardians of the study subject to collect information regarding any illness that had occurred since the last visit, symptoms experienced, use of healthcare facilities and use of medicines.

### Laboratory methods

Thick smears were prepared in duplicate so that if the subject had symptoms of malaria, one smear could be stained with Field's stain and read promptly to guide treatment. The other smear was stained with Giemsa stain and 200 high power fields (HPF) were examined before a smear was declared negative. Only the Giemsa-stained slide readings were used for the trial analysis. Parasite density was expressed per µl with the assumption that 1 parasite per high-powered field (hpf) equals 500 parasites per µl [10]. All slides were read by two laboratory technicians. If there was disagreement between their readings on parasite positivity or if the difference in the log-densities recorded was more than 1.5, slides were read by a third technician. Agreement was reached among the three microscopists after the slides had been re-checked. Discrepancies occurred mainly in smears with very low parasite densities. During follow-up, when a laboratory technician was not available to read a thick blood smear for malaria parasites, the Core™ Malaria Pf test (CORE Diagnostics, Birmingham, UK) was used to guide treatment and, a thick blood smear was collected for subsequent confirmation of the diagnosis.

Haemoglobin concentration was measured at recruitment, during morbidity surveillance and at the end of malaria transmission season surveys using a portable haemoglobinometer (HemoCue AB, Sweden).

### Statistical analysis

The primary study endpoint was the proportion of children who reported an adverse event during at least one of the three follow up surveys. Secondary endpoints were the number of clinic attendances with malaria during the surveillance period and the mean Hb concentration and the prevalence of asexual parasitaemia recorded in the cross-sectional survey in December. Sample size was calculated for comparison of each treatment group to the group that received SP plus AQ, the best regimen identified in a previous trial conducted previously in Niakhar, Senegal [5]. Based on previous experience, we assumed that the frequency of adverse event in the SP plus AQ group would be 15 to 20%. A trial with 286 children per arm has over 80% power to detect a halving in the frequency of adverse events, using a significance of 0.025 (to preserve an overall type 1 error rate of 5% for the primary endpoint when there are 2 comparisons, each alternative drug group to be compared with SP plus/ AQ). Based on experience from several previous studies, the drop-out rate was not expected to exceed 15%. Allowing for a 15% drop out, a sample size of 286×3/(1−0.15) = 1009 was therefore needed.

The primary analysis included all individuals who were allocated to treatment provided and who were followed up at the three visits that they received at least one follow-up visit, regardless of the number of treatments received. Data were analysed according to the treatment group to which a child was allocated. The prevalence of each type of adverse event was computed for the different treatment and control groups and presented with an exact binomial confidence interval. Fisher's exact test was used to test for differences in prevalence between the treatment groups. The overall frequency of adverse events was computed separately for each month and a Chi-square test for trend was used to test for a time trend in the prevalence of adverse events.

For the secondary analysis, malaria incidence was estimated for the treatment and control groups. To do this we calculated the total child years of follow-up in each group and the number of first cases of malaria. Rates were calculated with exact confidence intervals based on the Poisson distribution. For rate ratios, exact Poisson regression was used to obtain confidence intervals. All treated cases with parasitaemia at any density were included in the analysis of efficacy. Differences in mean Hb concentrations between treatment groups in December were estimated using analysis of covariance, adjusting for the Hb measurement at enrolment. All analyses were done with STATA software version 10 (College Station, TX, USA).

### Ethical review

The study was approved by the London School of Hygiene & Tropical Medicine ethics committee and by the joint MRC/Gambia Government ethics committee.

## Results

**Figure 1:**
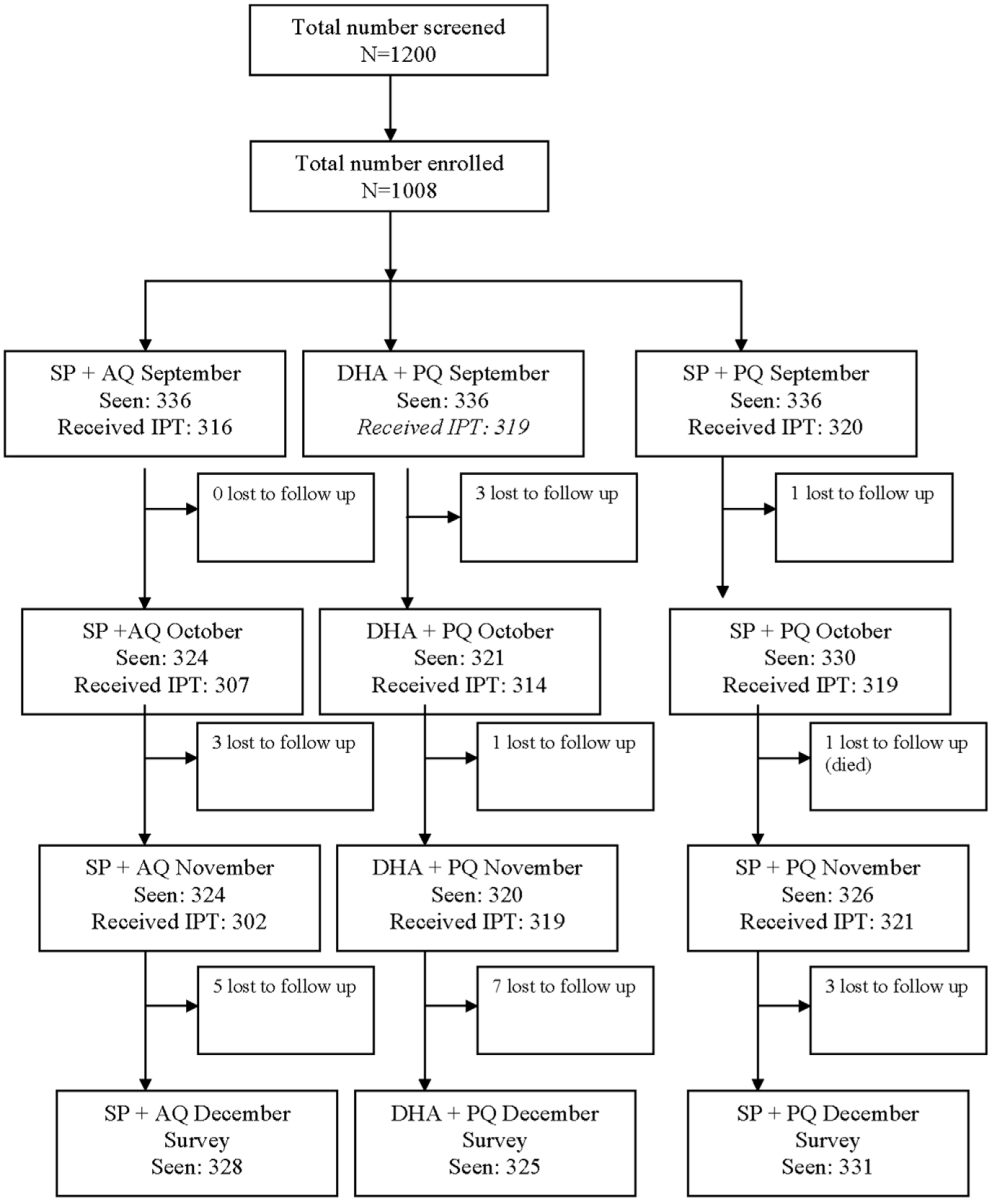
Trial profile.

One thousand two hundred children aged 6–59 months were screened and allocated to receive monthly IPT with SP plus AQ, SP plus PQ or DHA plus PQ. One thousand and eight children (84%) who were enrolled, treated and followed up for at least one visit were included in the primary analysis. The first dose of IPT treatment was given between 13–24 September 2007, the second dose between 16–26 October and the final dose between 13–24 Novembers. A cross-sectional survey was carried out between 6–13 December 2007. One of these 1008 children died in a road traffic accident and a further 23 were lost to follow up before the end of the surveillance period (Figure 1). Loss to follow up was similar among the treatment groups and was not associated with any of the baseline characteristics (Table 1). Among the 1008 included in the analysis, 331, 328, and 331 children received at least 2 treatments of SP plus AQ, SP plus PQ and DHA plus PQ respectively (Table 2). At enrolment the three treatment groups were similar with respect to variables such age, bednet usage, mean weight or mean haemoglobin concentration (Table 3). *Plasmodium falciparum* parasitaemia was found infrequently at enrollment.

**Table 1:** Loss to follow up by baseline characteristics and treatment group.

	N	No.	%	
Age (months)				
<12	6	0	0	
12–23	160	4	2.5	
24–35	268	4	1.5	
36–47	248	6	2.4	
48+	318	7	2.2	
Total	1000	21	2.1	P = 0.876
Weight (Kg)				
6–10	198	4	2	
10–14	550	11	2	
14–18	242	6	2.5	
18–22	15	0	0	
Total	1005	21	2.1	P = 0.967
Asexual parasitaemia				
No	992	21	2.1	
Yes	13	0	0	
Total	1005	21	2.1	P = 0.759
Hb <9g/dL				
<9	229	3	1.3	
> = 9	767	18	2.3	
Total	996	21	2.1	P = 0.439
ITN usage				
Yes	683	11	1.6	
No	322	10	3.1	
Total	1005	21	2.1	P = 0.155
Treatment group				
SPplusAQ	336	8	2.4	
DHAplusPQ	336	11	3.3	
SPplusPQ	336	5	1.5	
Total	1008	24	2.4	P = 0.338

**Table 2:** Number of doses of trial medication received and compliance.

	SPplusAQ (n = 336)		DHAplusPQ (n = 336)		SPplusPQ (n = 336)	
	No.	%	No.	%	No.	%
No. first doses completed						
0	0	0	1	0.3	1	0.3
1	5	1.5	7	2.1	4	1.2
2	73	21.7	39	11.6	37	11
3	258	76.8	289	86	294	87.5
Home dose taken (Sept)						
No	51	15.2	25	7.4	40	11.9
Yes	285	84.8	311	92.6	296	88.1
Home dose taken (Oct)						
No	57	17	37	11	43	12.8
Yes	279	83	299	89	293	87.2
Home dose taken (Nov)						
No	57	17	27	8	29	8.6
Yes	279	83	309	92	307	91.4

**Table 3:** Baseline characteristics at enrolment by treatment group.

	SP plus AQ		DHA plus PQ		SP plus PQ	
	No.	%	No.	%	No.	%
Age (months)						
<12	1	0.3	3	0.9	2	0.6
12–23	56	16.8	51	15.3	53	15.9
24–35	95	28.5	86	25.8	87	26
36–47	78	23.4	86	25.8	84	25.1
48+	103	30.9	107	32.1	108	32.3
Total	333	100	333	100	334	100
Age (mean, range)	38.79 (0,69)		39.35 (0,72)		39.43 (5,68)	
Weight ( Kg)						
6–10	79	23.6	59	17.6	60	17.9
10–14	178	53.1	189	56.4	183	54.6
14–18	72	21.5	81	24.2	89	26.6
18–22	6	1.8	6	1.8	3	0.9
Total	335	100	335	100	335	100
Weight (mean, SD)	12.12 (2.61)		12.36 (2.56)		12.36 (2.57)	
Asexual parasitaemia						
No	333	99.4	327	97.6	332	99.1
Yes	2	0.6	8	2.4	3	0.9
Total	335	100	335	100	335	100
Hb <9g/dl						
Yes	81	24.3	63	19	85	25.6
No	252	75.7	268	81	247	74.4
Total	333	100	331	100	332	100
Hb (mean,SD)	10.09 (1.65)		10.39 (1.69)		10.13(1.71)	
ITN usage						
Yes	221	66.0	228	68.1	234	69.9
No	114	34.0	107	31.9	101	30.1
Total	335	100	335	100	335	100

*The mean age of children in the control group was 37.7 months (range :12, 68) and 5%, 20.1%, 23.8%, 25.2% and 30.4% were in the <12 months, 12–23 months, 24–35 month, 36–47 months and 48+ months age groups respectively.

### Compliance with IPTc

Table 2 shows the number of children who came for their scheduled monthly treatment and the corresponding compliance during the malaria transmission period. The number of children who received the first dose of chemoprevention at the health centre was similar in the three treatment groups. However, the number of children who completed all the three doses was less in the SP plus AQ treatment group (77%) compared to the SP plus PQ (87.5%) and DHA plus PQ (86%) groups (chi-square = 16.4, df = 2, p-value<0.001). In addition, more children in the SP plus AQ group failed to complete their monthly treatment at home compared to those in the SP plus PQ and DHA plus PQ groups. (September, chi-square = 9.96, 2df, P = 0.007; in October, chi-square = 52.2, 2df, P<0.001; in November, chi-square = 16.8, 2df, P = <0.001).

### Safety and tolerability

Field workers visited study subjects three days after the start of each treatment round to document the incidence of adverse reactions. A summary of solicited adverse events is provided in Table 4. Monthly treatment with the three treatment regimens was generally well tolerated. No severe adverse reactions attributable to the study drugs, such as anaphylaxis, were detected in any volunteer during the study period. No severe skin reaction was reported in any child at any stage of the trial. The most frequent adverse events reported were coughing, diarrhoea, vomiting, loss of appetite and abdominal pain. Coughing was present in 15.2%, 15.4% and 18.7% of study subjects who received SP plus AQ, DHA plus PQ and SP plus PQ respectively, compared to 18.9% in the control group. 13.3%, 13.2%, and 10.3% of children in the SP plus AQ, DHA plus PQ and SP plus PQ groups respectively developed diarrhoea, compared to 17.5% in the control group. Loss of appetite was present in 8.2%, 6.4% and 7.2% of children in the SP plus AQ, DHA plus PQ and SP plus PQ groups respectively, compared to 13.1% in the control group. In general, symptoms were more common in the control group than in the three treatment groups. No grade 3 adverse events were reported in any treatment group during the follow-up period. Three children in the DHA plus PQ and one child in SP plus AQ group were admitted to hospital because of an acute respiratory infection. One child in the SP plus PQ group developed a febrile convulsion. One child in the SP plus AQ group was treated for severe dehydration. Only one child, who was in the DHA plus PQ group, died as a result of a road traffic accident.

**Table 4:** Percentage of children with a solicited adverse event during one or more of the treatment rounds.

	SPplusAQ (n = 329)		DHAplusPQ (n = 325)		SPplusPQ (n = 331)		Controls (n = 286)		
Symptom	No.	% (95% CI)	No.	% (95% CI)	No.	% (95% CI)	No.	% (95% CI)	*p-value
Abdominal pain	22	6.5(4.1,9.7)	21	6.3(3.9,9.5)	19	5.7(3.4,8.7)	36	12.5(8.9,16.9)	0.89
Diarrhoea	45	13.4(9.9,17.5)	44	13.2(9.7,17.3)	34	10.1(7.1,13.9)	50	17.4(13.2,22.3)	0.34
Drowsiness	2	0.6(0.1,2.1)	1	0.3(0,1.7)	2	0.6(0.1,2.1)	4	1.4(0.4,3.5)	1.00
Headache	9	2.7(1.2,5)	9	2.7(1.2,5.1)	14	4.2(2.3,6.9)	23	8.0(5.1,11.8)	0.49
Itching	14	4.2(2.3,6.9)	15	4.5(2.5,7.3)	17	5.1(3.0,8.0)	21	7.3(4.6,11)	0.86
Jaundice	5	1.5(0.5,3.4)	2	0.6(0.1,2.1)	3	0.9(0.2,2.6)	3	1.0(0.2,3.0)	0.62
Loss appetite	28	8.3(5.6,11.8)	22	6.6(4.2,9.8)	25	7.4(4.9,10.8)	37	12.9(9.2,17.3)	0.69
Malaise	9	2.7(1.2,5)	9	2.7(1.2,5.1)	11	3.3(1.6,5.8)	15	5.2(3.0,8.5)	0.92
Nausea	7	2.1(0.8,4.2)	7	2.1(0.8,4.3)	6	1.8(0.7,3.8)	10	3.5(1.7,6.3)	0.96
Skin rash	10	3(1.4,5.4)	10	3(1.4,5.4)	15	4.5(2.5,7.3)	26	9.1(6.0,13.0)	0.52
Sleep disorder	4	1.2(0.3,3)	7	2.1(0.8,4.3)	9	2.7(1.2,5.0)	12	4.2(2.2,7.2)	0.39
Vomiting	19	5.7(3.4,8.7)	17	5.1(3.0,8.0)	25	7.4(4.9,10.8)	38	13.2(9.5,17.7)	0.43
Cough	51	15.2(11.5,19.5)	51	15.3(11.6,19.6)	63	18.8(14.7,23.3)	55	19.2(14.8,24.2)	0.38
Any	129	38.4(33.2,43.8)	134	40.1(34.8,45.6)	141	42(36.6,47.4)	158	55.1(49.1,60.9)	0.65

*p-value from a comparison of the three treatment groups using Fisher's exact test.

% refers to the proportion of children who reported an adverse event on any occasion after drug administration.

To test whether the risk of reported adverse events varied with the number of doses received, separate analyses were carried out for each of the treatment rounds and the results showed significant decrease in the risk of adverse events in the SP plus PQ group when measured after the 2nd and 3rd dose compared to the first round (Table 5).

**Table 5:** Frequency of adverse events by course of treatment.

	SPplusAQ		DHAplusPQ		SPplusPQ		Control	
	N	% (95% CI)	N	% (95% CI)	N	% (95% CI)	N	% (95% CI)
Sept	61/329	18.5 (14.5,23.2)	62/325	19.1 (15.0,23.8)	72/331	21.8 (17.4,26.6)	77/286	26.9 (21.9,32.5)
Oct	49/324	15.1 (11.4,19.5)	47/316	14.9 (11.1,19.3)	52/328	15.9 (12.1,20.3)	74/280	26.4 (21.4,32.0)
Nov	50/323	15.5 (11.7,19.9)	58/320	18.1 (14.1,22.8)	47/326	14.4 (10.8,18.7)	67/275	24.4 (19.4,29.9)
P-value*	0.29		0.745		0.013		0.491	

% refers to the proportion of children who reported any adverse event.

*P-value based on a score test for trend.

### Malaria incidence

The incidence of clinical attacks of malaria was generally low in all the treatment groups; overall incidence of malaria among children in the trial was 0.22 per child year (95% CI 0.16, 0.28). No child had more than one episode of malaria. The incidence of malaria in the DHA plus PQ, SP plus AQ and SP plus PQ groups was 0.10 (0.05, 0.22), 0.06 (0.022, 0.155) and 0.06 (0.02, 0.15) respectively. The incidence of malaria in the non-randomized control group measured by passive case detection was 0.79 (0.58, 1.08) [Tables 6 and 7]. The percentage reduction in incidence compared to the control group was 87% (95%CI 71%, 95%) for DHA plus PQ, 93% for SP plus AQ and 93% (80%, 98%) for SP plus PQ).

**Table 6:** Incidence of malaria by treatment group (any parasitaemia).

	Cases	Child years	Rate (95% CI)
Control	41	51.81	0.79 (0.57–1.07)
SP plus AQ	4	68.81	0.06 (0.02–0.15)
DHA plus PQ	7	67.84	0.10 (0.04–0.21)
SP plus PQ	4	69.60	0.06 (0.02–0.15)

**Table 7:** Incidence of malaria by treatment group (parasitaemia ≥5000/µl).

	Cases	Child years	Rate (95% CI)
Control	34	52.47	0.65 (0.45–0.91)
SP plus AQ	2	69.04	0.03 (0.00–0.10)
DHA plus PQ	6	68.04	0.09 (0.03–0.19)
SP plus PQ	0	70.17	0.00 (0.00–0.05)

### Cross-sectional survey at the end of the malaria transmission season

Ninety-seven percent (982/1008) of the study children (328 SP plus AQ, 324 DHA plus PQ, 330 SP plus PQ) were seen during the end of the rainy season survey when blood samples for determination of parasitaemia and haemoglobin were collected. The prevalence of parasitaemia was very low (0.3% SP plus AQ, 2.2% DHA plus PQ, 0.6% SP plus PQ). Mean Hb concentration was similar in the three groups (10.1 g/dL SP plus AQ, 10.4 g/dL DHA plus PQ, 10.1 g/dL SP plus PQ) [Table 8]. Moderate anaemia was present in 4%, 5% and 7% of the children in SP plus AQ, DHA plus PQ and SP plus PQ treatment groups respectively. Four children were severely anaemic (Hb <5 g/dL) at the time of the December survey, two in SP plus PQ group and one each in SP plus AQ and DHA plus PQ groups.

**Table 8:** Haematological findings at the end of transmission season cross-sectional survey (December).

	SPplusAQ		DHAplusPQ		SPplusPQ	
	(n = 328)		(n = 324)		(n = 330)	
	Estimate	95% CI	Estimate	95% CI	Estimate	95% CI
Mean	10.08	9.88,10.28	10.35	10.15,10.55	10.13	9.92,10.33
*Adjusted mean difference	0		0.07	−0.14,0.28	0.04	−0.17,0.25
P-value (T-test)			0.519		0.718	
% Moderate Aneamia (<7g/dL)	0.04	0.02,0.06	0.05	0.03,0.08	0.07	0.04,0.09
RR	1		1.32	0.65,2.68	1.68	0.86,3.28
P-value (Chi-square)			0.43		0.12	

*Adjusted for Hb measurement at enrolment.

## Discussion

In this study, we have compared the safety, tolerability and efficacy of three potential drug combinations for intermittent preventive treatment of malaria in children. All three treatment regimens investigated, including the one used in the previous Senegalese trial (SP plus AQ), were safe and efficacious. The incidence of malaria during the trial and the prevalence of parasitaemia at the end of the malaria transmission season were very low in all the three treatment groups. These results are consistent with findings from previous trials of IPTc carried out in other West African countries which showed significant levels of protection against clinical attacks of malaria during the malaria transmission season [3], [4], [6]. Overall, 68% of the study subjects used insecticide-treated bednets (ITNs), and it is possible the widespread use of ITNs contributed to the low incidence of clinical attacks of malaria observed in this trial.

All three combination investigated were safe. No serious adverse event attributable to study medication was observed. Minor adverse events observed in this trial occurred infrequently in all the treatment groups and were similar in the three treatment groups. Minor adverse events were more common in the control group than in the three treatment groups and these were very likely due to common childhood illnesses such as malaria as the incidence of malaria was higher in the control group compared to the three treatment groups. Previous studies of IPTc in Senegal suggested that significantly more minor adverse events occur in children who received amodiaquine-containing preparations than in those who received artesunate and SP [5]. In contrast a study in Ghana showed that the incidence of mild adverse events was similar in the placebo and treatment group including those who received amodiaquine-containing preparations [6]. These conflicting results emphasise the need to monitor adverse events if drugs are be used on a large scale for IPT.

AQ has been used widely for treatment of uncomplicated malaria in the past and is now used in combination with artesunate as first line treatment for malaria in several countries in Africa with no apparent problems over safety. A review of the use of AQ for treatment of malaria in Africa concluded that it is safe [11]. Measurements of white blood cell count and liver function were not done to determine the safety of AQ in this study as the serious adverse effects that have been associated with the prophylactic use of AQ (agranulocytosis and severe liver toxicity) are considered rare when it is used for malaria treatment [12]. The principal active metabolite of amodiaquine has a long half-life (9–18 days) and this makes it a desirable drug for IPT [13].

SP has been used widely for IPT in infants and in pregnant women because of its long half-life, safety, wide availability, low-cost, ease of delivery (a single dose treatment) and a good acceptability profile. The main concern about the use of SP for IPT is the propensity for this drug to cause severe skin reactions in a small proportion of subjects. No adverse cutaneous events were recorded in children in the current study. The main threat to the use of SP in IPT regimens comes from increasing resistance to SP, especially in East Africa. Thus, there is a need to find alternative drugs for chemoprevention which should, ideally, be used in combination to reduce the likelihood of the emergence of resistance.

As there is now increasing evidence that IPT works in infants and children through its prophylactic effect [14], [15] any replacement for SP needs to be long acting, the aim in the case of seasonal IPTc being to provide a period of chemoprophylaxis during the time of the year when the risk from malaria is greatest. [14], [15]. Thus, if the duration of suppressive chemoprophylaxis is an important determinant of the efficacy of IPT, drugs with long-half lives are likely to be more effective. Drugs to be used for IPT should have a good safety profile, be well tolerated to improve compliance and acceptability, preferably given as single dose treatment and affordable.

Piperaquine is attractive as a candidate drug for IPT as it is safe, with a toxicity profile similar to that of chloroquine, and an estimated terminal elimination half-life of approximately 17 days. The combination of piperaquine with SP is a logical one for IPT as the two drugs have long half lives. However, currently there is no good manufacturing practice (GMP) product of piperaquine available for general use. Dihydroartemisinin-piperaquine is an artemisinin-containing, fixed-combination antimalarial drug, developed in China, which is safe and highly effective in the treatment of clinical malaria [16]. However, it is not an ideal combination for chemoprevention because of the very short half- life of the artemisinin component, resulting in a significant period in which new infections are exposed to a single drug. Mefloquine is a possible candidate for IPT as it has a long half-life, can be given as a single or split dose and because resistance to mefloquine is not a significant problem in Africa. It is used for chemoprophylaxis in short-term non-immune visitors to endemic countries and is effective when used for IPT in pregnant women [17]. However, a recent study conducted in Tanzania, showed that mefloquine was effective when used for IPT in infants but caused vomiting [18]. Mefloquine is relatively expensive and it has not yet been evaluated for IPT in older children. In the Gambia, SP plus AQ and SP plus PQ were both highly effective and safe regimens for IPTC but this might not be the case in countries where the prevalence of SP resistance is higher than in The Gambia.

For IPTc to be an effective malaria control tool, a reasonable level of compliance with drug-taking must be maintained with minimum outside support. Whether this can be achieved will depend upon the attitude of both the health authorities who administer the drug and the mothers/guardians who take their children to the health centre or to the community worker to receive the tablets. In the current study, compliance was generally good for all the treatment regimens. However the number of children who completed all the three doses was significantly less in the SP plus AQ treatment group compared to the SP plus PQ and DHA plus PQ groups. It is not clear if the lower compliance was due to the reduced tolerability of AQ seen in other studies as the frequency of minor adverse events observed in all the treatments groups was similar.

A potential weakness of the trial was that it was not a placebo-controlled trial, the optimum design for most clinical trials, but this approach was not considered desirable because a trial conducted along these lines would have resulted in some children not receiving IPTc. As IPTc has been shown previously to reduce morbidity from malaria, it was considered ethically unacceptable to include a control group. The possibility of unobserved selection bias and confounding cannot be ruled and the results provided on the differences in the incidence of clinical episodes of malaria in the treatment groups compared with the control group must be treated with caution. However, it appears that all treatment groups were highly efficacious.

IPT in infants and pregnant women has a major advantage over IPT in older children in that a delivery system for drug administration, the reproductive and child health clinic (RCH) already exists. In contrast, the optimum delivery strategy for IPTc is not known. A number of studies are under way to identify ways in which this highly efficacious intervention could be most effectively delivered.

There may be other groups of children who are at particular risk from malaria and anaemia who could benefit from IPT. These include children with sickle cell disease or HIV infection. In older children, seasonal IPT might improve school attendance and performance. Thus, there is a need to investigate whether SP plus AQ, SP plus piperaquine (PQ) and dihydroartemisinin (DHA) plus PQ might be equally safe and effective when used for IPT in these groups of children who are at particular risk of malaria.

## Supporting Information

Protocol S1 (0.17 MB DOC)

Checklist S1 CONSORT checklist.
(0.05 MB DOC)
