# Supplementary material for: A Randomised Trial to Compare the Safety, Tolerability and Efficacy of Three Drug Combinations for Intermittent Preventive Treatment in Children
Source: PLoS One. 2010 Jun 21;5(6):e11225. doi: 10.1371/journal.pone.0011225 (PMC2888611; doi:10.1371/journal.pone.0011225)
Supplement: Protocol S1 — (0.17 MB DOC) [file pone.0011225.s001.doc]

**TITLE OF PROPOSAL**

A randomised trial to compare the safety, tolerability and efficacy of three potential drug

combinations for intermittent preventive treatment in children aged 1 to 5 years in an area of seasonal malaria transmission in Upper River Region, The Gambia

**Summary**

Intermittent preventive treatment (IPT) offers a potential way of preventing malaria infection without compromising the development of malaria immunity or encouraging drug resistance. The effect of IPT in children in the prevention of malaria has been evaluated in a number of trials in areas of seasonal malaria transmission. Results from these trials have shown that IPTc provided between 40% - 86% protection against clinical malaria. In 2006, a trial that compared two methods of IPTc delivery was carried out in Upper River Division, The Gambia. Preliminary results of the trial have shown that the treatment was very effective as only 4% (45/1133) of the children seen at the end of year cross-sectional survey were parasitaemic. Tolerability was assessed in a subset of 1100 children and the results showed that about 13.5% of children developed mild to moderate vomiting. Malaise was present in about 10% of the study subjects. Severe adverse events were rare. Thus it is important to investigate if other drug regimens might be equally effective in preventing malaria but less likely to cause adverse events. During the 2007 malaria transmission season, 1009 children aged 1-5 years will be individually randomized to receive amodiaquine plus SP, piperaquine plus SP or Artekin ^TM^ (dihdroartemisinin plus piperaquine) at monthly intervals on three occasions during the months of September, October, and November. To determine the prevalence of side effects following drug administration participants in each treatment group will be visited at home three and seven days after each round of drug administration and a side effects questionnaire completed. To help establish whether these adverse events are drug related, the same questionnaire will be administered after each treatment round, to 286 age-matched children who are not part of the trial. The primary ends points will be the incidence of adverse events.

1.0 Background

*1.1 Malaria control in Sub-Saharan Africa*

Although tremendous advances have been made in controlling malaria during the last two decades, the disease remains a major killer in sub-Saharan Africa. Malaria is estimated to cause over one million deaths a year and the vast majority of deaths occur among young children, especially in remote rural areas of Africa (WHO 2000). Community based trials with insecticide-treated bednets (ITN) , which started in the late 1980s in The Gambia and elsewhere, achieved a 40 to 60% short term-reduction in malaria mortality in children less than 5 years in areas with high coverage (Alonso et al. 1993; Greenwood and Pickering 1993). However, achieving high levels of coverage with ITN has proved difficult and other control strategies are needed. Anti malarial chemoprophylaxis for the whole paediatric population has been shown to reduce malaria morbidity and all cause mortality but this strategy is not generally encouraged due to the threat of resistance and difficulties in implementation (Laing , 1984; Pringle and Avery-Jones, 1966; Greenwood, 2004). Chemoprophylaxis can also result in loss of acquired immunity or a delay in the development of clinical immunity leading to a period of increased risk of clinical malaria on cessation of the intervention. Intermittent preventive treatment offers a potential way of preventing malaria infection without compromising the development of malaria immunity or encouraging drug resistance. Use of drugs in combination for IPT may help to prevent selection for resistance.

*1.2. Intermittent preventive treatment*

*1.2.1 Definition*

IPT can be defined as drug administration, in curative doses, given at specific opportunities (for example ante-natal visits or routine vaccinations), with the objective of preventing malaria. Drugs are provided irrespective of the presence of parasites or symptoms. Drugs used for IPT must be very safe as well as effective.

*1.2.2 Intermittent preventive treatment in infants (IPTi)*

The concept of IPT was initially developed for the control of malaria in pregnancy and the administration of SP on two occasions during the second and third trimesters of pregnancy is now recommended practice in areas of medium or high malaria risk where SP resistance is still uncommon (Parise et al., 1998; Shulman et al., 1999). This approach was subsequently adapted to the prevention of malaria in Tanzanian infants. In a study in Tanzania in infants, three doses of SP given at the time of vaccinations with DPT2, DPT3 and measles vaccine reduced the rate of clinical malaria by 59% and that of severe anaemia by 50% during the first year of life in an area of intense transmission (Schellenberg et al., 1997). Significantly, this protective effect persisted up to the age of 24 months (Schellenberg et al., 2005). In a trial conducted in another part of Tanzania, IPT with amodiaquine provided 65% protection against malaria fevers and 67% protection against anaemia without evidence of subsequent rebound (Massaga et al., 2003). However, different results were obtained in a study undertaken in northern Ghana where malaria transmission is intense and seasonal. Significant levels of protection against clinical attacks of malaria (25%) and episodes of anaemia (35%) were observed during the first year of life but more episodes, especially those associated with high density parasitaemia, occurred in children who had received IPT than in controls during eight months period which commenced three months after the last dose of IPT had been given (Chandramohan *et al.,* 2005). Overall, IPT provided only 12% protection against episodes of anaemia during the first two years of life. In a more recent trial conducted in southern Mozambique, an area with perennial transmission, IPTi during the ﬁrst year of life reduced the incidence of clinical malaria by 22 % and the rate of hospital admissions by 19%. However, the intervention did not have any significant effect on the incidence of severe anaemia (packed cell volume of < 25%) [Macete *et al.,* 2006] . Results from two further trials conducted in Ghana (submitted for publication) have given results similar to those found in the study of Chandramohan et al. (2005), about 25% protection against clinical attacks of malaria. No other trials have observed the sustained protection seen in the first study conducted in Tanzania and in each of the more recent studies conducted in Ghana there has been some suggestion of a rebound in the year after treatment was give. Evidence from the more recent trials suggests that the effectiveness of SP in IPTi depends upon its chemoprophylactic effect.

***1.2.3 IPT in children (IPTc).***

In areas of intense malaria transmission a high proportion of the mortality and severe morbidity due to the infection occurs during infancy. IPTi could have a significant impact on overall malaria morbidity in childhood in such communities. In contrast, in areas of seasonal transmission, the major burden of malaria is not in infants but in older children. In such areas, IPTi alone is not likely to have a major impact on the overall burden of malaria. For IPT to have a significant impact on malaria mortality and morbidity in such areas it needs to be given to children as well as infants.

The role of IPTc in the prevention of malaria and anaemia has been evaluated in a limited number of trials. In Mali, an area of seasonal malaria transmission, two doses of SP given to children aged 6 months to 9 years at an interval of two months gave a protective efficacy of 40% against clinical attacks of malaria among children in the intervention group compared to the placebo group (Dicko et al, 2004). In another, study in Niakhar, Senegal IPTc in an area of intense but short seasonal malaria transmission, SP and one dose of artesunate given to children less than 5 years old three times at one monthly interval throughout the peak period of malaria transmission season resulted in an 86% reduction in clinical malaria (Cissé B et al, 2006). Thus, these results suggest that IPTc has potential as an affordable malaria and anaemia control tool in areas of seasonal transmission.

It is not certain whether IPTc could be effectively delivered through the infrastructure of the Reproductive and Child Health (RCH) service given the ever increasing workload of the RCH clinics. With support from the Gates Malaria Partnership, a study to compare two strategies for the delivery of intermittent preventive treatment in children was undertaken in 2006 in 14000 children in Upper River Division of The Gambia where malaria transmission is seasonal. Twenty-six RCH trekking clinics in Upper River Division were randomly allocated to receive IPT from the RCH trekking team or from a village health worker. Treatment with a single dose of sulfadoxine /pyrimethamine (SP) plus three doses of amodiaquine was given to all study subjects at monthly intervals on three occasions during the months of September, October and November. Preliminary analysis of the results showed that the treatment was very effective as only 4% (45/1133) of the children seen at the end of year cross-sectional survey were parasitaemic. Compliance was initially high but decreased progressively with increase in the number of doses taken by the study subjects. 81%, 74% and 67% of study subjects in the VHW arm received IPT during September, October and November respectively. In the RCH arm, 69%, 51% and 48% received IPT during September, October, and November respectively. Tolerability was assessed in a subset of 1100 children and the results showed that about 13.5% of children developed mild to moderate vomiting but there was no control group for comparison. Mild to moderate malaise was present in about 10% of the study subjects. Severe adverse event was rare. However, these adverse events had a negative impact on compliance because of the perception among the study population that the trial medication was associated with a significant number of adverse events. Thus, it is important to investigate if other drug combinations might be equally effective in preventing malaria but more effective in preventing adverse events. Thus, we now propose to compare the safety, efficacy and tolerability of three potential drugs combinations for IPTc. The drugs combinations that we have selected for comparison are amodiaquine plus sulfadoxine-pyrimethamine (the combination used in the previous study), sulfadoxine-pyrimethamine plus piperaquine (another long acting drug) and Artekin ^TM^ (dihydoartemsinin plus piperaquine).

*1.2.4 Potential drugs for use in IPT*

Amodiaquine

Amodiaquine (AQ) is a 4-aminoquinoline antimalarial drug similar in structure and activity to chloroquine with antipyretic and anti-inflammatory properties. It is rapidly converted to the metabolite DEAQ and the antiparasitic effect is mainly attributed to this metabolite. A review of published and unpublished studies conducted in Africa on the treatment of uncomplicated falciparum malaria showed that AQ is significantly more effective than chloroquine (CQ) in clearing parasites, with a tendency for faster clinical recovery (Olliaro et al., 1996). The most common adverse reactions to AQ when it is given in therapeutic doses for malaria treatment are nausea, vomiting, abdominal pain, diarrhoea and itching and a less common effect is bradycardia (Brasseur et al., 1995, Sowunmi et al., 2001). When used for chemoprophylaxis in Caucasians, AQ caused toxic hepatitis and fatal agranulocytosis. The incidence of serious reactions in UK travellers using AQ for prophylaxis was 1 in 1 700. Blood disorders occurred in 1 in 2 200 travellers and serious hepatic disorders in 1 in 15 650. Fatal events occurred in 1 in 15 500 travellers (Phillips-Howard et al., 1990). However, AQ has been used extensively in Africa for the treatment of malaria, especially in francophone Africa, with few reports of serious adverse effects.

Sulphadoxine-pyrimethamine

Sulphadoxine-pyrimethamine (SP) is an antifolate combination drug, which acts by synergism against the parasite-specific enzymes, dihydropteroate synthetase and dihydrofolate reductase. It is an effective blood schizonticide against *P. falciparum* but less effective against other species of plasmodium. It does not show cross-resistance with the 4-aminoquinolines, mefloquine, quinine, halofantrine or the artemisinin derivatives. It has a long half-life, hence its formulation as a single dose drug, which is why compliance to it is high. SP has been used successfully in areas with highly developed *P. falciparum* resistance to chloroquine and during malaria epidemics. However, the long half-life of SP provides a potent selective pressure for parasite resistance in areas of high transmission. Therapeutic doses of SP for malaria are generally well tolerated. The most serious adverse reactions are associated with hypersensitivity to the sulphadoxine component, involving the skin and mucous membranes, normally occurring after repeated administration. A high incidence of cutaneous side effects precluded the use of SP for prophylaxis in Caucasian travellers. However serious cutaneous reactions following single-dose treatment with SP are rare and analysis of the large data base on the safety of SP when used for IPTi carried out by the IPTi Consortium has shown that SP is very safe when used for IPT in infants.

Amodiaquine/sulphadoxine-pyrimethamine (AQ/SP) combination

A systematic review and meta-analysis of seven randomised trials on the comparative efficacy and safety of AQ plus SP and artemisinin-based combinations for the treatment of uncomplicated falciparum malaria in African children showed that treatment failures were significantly fewer following treatment with AQ plus SP than after treatment with artesunate (AS) plus SP but were increased compared with Coartem, and similar compared with AQ plus AS. The incidence of serious adverse events was similar in all the treatment arms, although the numbers were small (Obonyo et al 2006). A trial undertaken in Senegal in 2004 children compared different treatment regimens for IPTc . The combinations investigated were SP plus one dose of artesunate, SP plus three doses of artesunate, SP plus three doses of AQ and three doses of AS and AQ. The children were visited after each treatment to record any adverse events and were kept under weekly malaria surveillance throughout the 2004 malaria season. The best results were obtained with SP plus AQ. This regimen showed the highest level of protection against infection and the lowest prevalence of parasitaemia at the end of the malaria transmission season. The main side effects recorded were fever, vomiting, agitation and headache. Each of these symptoms was seen more frequently in children who took SP plus AQ than in children in the SP plus AS groups, but the overall incidence was low (Sokhna et al. submitted).

Piperaquine

Piperaquine (PQP) is an orally active bisquinoline developed for clinical use in China in 1973(Wang et al., 1981, Yin et al., 1981, Yang et al., 1992, Lan et al., 1989, Chen et al., 1981, Shen et al., 1981). The phosphate salt is water-soluble. *In-vitro* testing in several laboratories has shown that PQP is approximately equivalent to CQ against sensitive parasites and significantly more effective than CQ against resistant *P.falciparum.* Like other members of this class of antimalarials, PQP chemically inhibits haem bio-crystallisation. The estimated terminal elimination half-life is approximately 17 days, but only limited pharmacokinetic (PK) data are available in man (Hung et al., 2003). The gastrointestinal side effects of PQP are similar to those of chloroquine but milder at total doses of less than 2g. Otherwise the toxicity profile is similar. PQP replaced CQ as recommended treatment for *falciparum* malaria in China in 1978. Over the next 14 years, 214 tons of PQP equivalent to 140,000,000 adult doses, were used for mass prophylaxis and treatment. Thus, in terms of drug treatment administered, PQP is amongst the most widely used antimalarial drugs in the world. Surveillance at the time found no adverse events other than rare cases of rash.

Dihydroartemisinin

Dihydroartemisinin (DHA) is the active metabolite of AS and artemether, and manufactured as an oral antimalarial drug in its own right in China. It has equivalent clinical efficacy to the more widely used artesunate. The mode of antimalarial action of this class of compounds is thought to involve intraparasitic Fe^++^ catalysed production of carbon centred free radicals. Recently, an alternative mode of action for the artemisinins has been reported involving irreversible inhibition of a metabolic enzyme - the malarial calcium-dependent ATPase (PfATP6). PfATP6 is very similar to a mammalian ATPase (the sarco/endoplasmic reticulum calcium-dependent ATPase, SERCA), which is located in the sarco/endoplasmic reticulum (ER) (Eckstein-Ludwig et al., 2003). DHA is absorbed and eliminated rapidly (t_1/2_ circa 45 min.). To date no significant resistance has been reported and stable high level resistance cannot be induced in the laboratory. These drugs are extremely safe in clinical use; there is no evidence that they produce neurotoxicity, or any other systematic toxic effects at therapeutic doses (Price et al., 1999, Looareesuwan et al., 1996). The only significant adverse effect is rare idiosynchratic type 1 hypersensitivity reactions (Leonardi et al., 2001). In experimental animals with early pregnancies, fetal toxicity leading usually to fetal resorption occurs with high doses of the artemisinin derivatives and some surviving embryos have congenital abnormalities. Clinical experience in pregnancy to date has shown no cause for concern (McGready et al., 2001). A recent WHO consensus committee concluded these drugs could be used in the second and third trimesters, but pending further information, not in the first trimester of pregnancy unless the life of the mother was at risk and no effective alternative was available(*WHO TDR/RBM 2002)*.

Artekin^TM^

Dihydroartemisinin-piperaquine (DP) is an artemisinin-containing, fixed-combination antimalarial drug developed in China. It has been evaluated extensively in clinical trials in Thailand, Vietnam, Cambodia, and China (Denis et al., 2002, Tran et al., 2004, Guo, 1993). Efficacy has been high and tolerability uniformly excellent in all trials in these multi-drug resistant areas including Cambodia and Vietnam (Denis et al., 2002, Tran et al., 2004), where PQP-resistance was common after extensive use. These trials have used a constant dose of PQP, but initially the co-formulation also included trimethoprim (CV8). This formulation (CV8) has been used extensively in Vietnam, where it is part of national policy. More recently the dose of DHA in DHA-PQP has been increased by 25%. This product (Artekin^TM^) has been registered in China, Cambodia and Vietnam and in several countries in Africa including Senegal. DP is being used increasingly in Southeast Asia and is already part of national treatment recommendations in Cambodia and Vietnam. An alternative formulation of Artekin ^TM^ is being developed by the Medicines for malaria venture and should be ready for licensure in 2008.

# *2.0 Objectives*

*2.1 Primary Objective*

To determine the safety and tolerability of AQ (three days) plus SP (one day), PQP (three days) plus SP (one day) and Artekin ^TM^ (PQP plus DHA) (3 days) when used for seasonal IPT in children.

*2.2 Secondary Objective*

To determine the efficacy of AQ (three days) plus SP (one day), PQP (three days) plus SP (one day) and Artekin ^TM^ (PQP plus DHA)(3 days) when used for seasonal IPT in children.

*3.0 Study design and methods*

*3.1 Study area*

The study will be based at the MRC Field Station in Basse in the Upper River Division (URD) of The Gambia. This is an area of savannah typical of much of sub-Sahelian Africa. Malaria transmission is seasonal, occurring mostly during the short rainy season (July - October), and is almost exclusively due to *P.* *falciparum*. *Anopheles gambiae* is the principal vector; the area has the highest entomological inoculation rate (EIR) in The Gambia (between 1 and 50 infective bites per person per year). The population has worked with the MRC for at least 20 years and cordial relations exist between the MRC and surrounding villages.

*3.2 Enumeration, recruitment and consent*

The study will be carried in collaboration with the National Malaria Control Programme and URD District Health Team and study children will be recruited from villages in the south bank of Upper River Division. During the preparatory phase of the study, the design and objectives of the study will be discussed extensively with the URD District Team and all health care providers working in the study area. The study team will visit all the villages in the study area to explain the objectives of the trial to village elders, opinion leaders and village health workers. This will be followed by village meetings, which the entire community will be invited to participate. During these meetings, agreement to participate in the study will be obtained from the villagers. In addition, parents or guardians of prospective study subjects in the right age group will be visited at home and they will be provided with flyers in English and the appropriate local language (Mandinka, Sarahule or Fula).

Written informed consent will be obtained from the parent(s) or guardian(s) before a child is enrolled in the study. Approximately 1400 children aged between 1 to 5 years will be screened, of whom 1009 volunteers who meet the enrolment criteria will be enrolled in the study to receive SP plus AQ, SP plus PQP or Artekin ^TM^. Clinic forms will be prepared and filled in by the study physician at screening. Each form will contain the subject’s initials, information about the subject’s date of birth, compound number, date of screening visit, whether the subject was enrolled or not and, when applicable, reasons for exclusion from the study based on medical history or physical or laboratory screening examination. A screening list will be prepared by the investigators as part of the routine source documentation. After review of the medical history, physical examination and laboratory results, reasons for non-eligibility will be documented on the list. Any clinically relevant finding will be treated at the discretion of the investigator. On entry into the trial a detailed clinical history, drug history, family history, and information on bednet usage and net impregnation will be obtained. The study physician will examine the child, document nutritional status and record the findings in a case report form. A finger-prick baseline blood sample will be obtained for determination of haemoglobin concentration and thick blood film preparation**.** Other investigations such as chest x-ray and urine examinations will be carried out as clinically indicated.

*3.3 Inclusion criteria*

1. Age between 1 to 5 years at enrolment.
2. Informed consent obtained from parents or legal guardians.
3. No current participation in another malaria intervention trial.
4. Free of obvious health problems as established by medical history and clinical examination before entering into the study.
5. Available for the duration of the study.

#

# *3.4 Exclusion criteria*

1. Known allergy to any of the antimalarial drugs used in the trial and if this is unknown, then a history of allergic reaction to any drug.
2. Acute or chronic, clinically significant pulmonary, cardiovascular, hepatic or renal functional abnormality, as determined by physical examination or laboratory tests.

*3.5 Withdrawal during the study*

***Withdrawal Criteria***

Study subjects may be withdrawn from the study for any one of the following reasons.

1. Withdrawal of parental / legal guardian’s consent.
2. Serious adverse event attributable to the study drug.
3. Loss to follow-up.

Children who develop adverse events attributable to study drugs should not receive further doses but should be followed-up to monitor safety up until the resolution of the adverse reaction.

*3.6 Randomisation*

Open-label, individual randomisation is proposed and will be done approximately three weeks before the first dose of IPTc administration planned for early September 2007. Study subjects will be individually randomised into either the AQ plus SP, PQP plus SP or Artekin^TM^ treatment groups in a 1:1:1 fashion. An independent statistician will generate the randomizing subject study number (SSN) that associates each SSN either to AQ plus SP, PQP plus SP or Artekin ^TM^ treatment groups and study subjects will be given treatment according to their randomisation group. A photo ID card will be given to all participants to facilitate identification at every contact, at home or in the health centres.

*3.7 Drug distribution at the health centres*

Study subjects and their parents will be informed about the date of treatment approximately one week before any intervention. Treatment will be given during the months of September, October and November. Drugs will be given at the nearest health centre by a designated member of staff. Tablets will be crushed, mixed with honey or suspended in water and given on a spoon. Staff who administer drugs at the health centre will play no further part in the trial. All participants will be kept under observation for 30 minutes after drugs are taken. In case of vomiting, the drug will be re-administrated. At the time of each monthly drug administration, a brief clinical assessment will be carried out. Children who are febrile will be screened for malaria using a rapid antigen test. Children found to have malaria will be treated with Lumefantrine-artemether (Coartem^TM^, Norvatis Pharma., Basel Switzerland).

*3.8 Treatment*

SP (tablets containing 500 mg sulfadoxine/ 25 mg pyrimethamine) will be given at an approximate dose of 1.25 mg pyrimethamine/25 mg sulfadoxine per kg stat.

Amodiaquine (200 mg base tablets) will be given at an approximate dose of 25 mg/kg over 3 days ( 10mg/kg D1, 10mg/kg D2 and 5mg/g D3).

PQ tablets (containing 320 mg per tablet) will be given at a target dose of 16.8 gm/kg once daily for 3 days.

Artekin *™* tablets tablet containing 40 mg of DHA and 320 mg PQ; Holleykin Pharmaceutical Co. Ltd., Guangzhou, China) will be used. Children will be given a dose of approximately PQP-DHA 1.6 / 12.8 mg/kg respectively. The drug will be given once daily for 3 days.

Coartem (tablet containing 20 mg artemether and 120 mg lumefantrine) will be given at the following doses: < 15kg: one tablet at the time of diagnosis, and then at 8, 24 and 48 hours.16-25kg : Two tablets as a single dose at the time of diagnosis, and then at 8, 24 and 48 hours.

*3.9 Assessment of adverse events*

# *3.9.1 Surveillance for adverse events*

An adverse event is defined as any unfavourable and unintended sign, symptom, syndrome, or illness that develops or worsens during the period of observation in the clinical study. Clinically relevant abnormal results of diagnostic procedures including abnormal laboratory findings (e.g., requiring unscheduled diagnostic procedures or treatment measures, or resulting in withdrawal from the study) which are considered by the Principal Investigator to be detrimental should be recorded as adverse events whether or not they have a causal relationship with the study drug. Where the laboratory result is a sign of another clinical condition, the clinical condition itself should be the reported adverse event. An event unequivocally caused by a significant deterioration of the underlying condition related to malaria is regarded as an adverse event.

To determine the prevalence of minor side effects following drug administration such as headache, fever, weakness, abdominal pain, anorexia, nausea, vomiting, diarrhoea, rash, itching or sleep disorder participants in each treatment group will be visited at home three and seven days after each round of drug administration and a side effects questionnaire completed. Field workers who complete these questionnaires will be unaware of the treatment group to which the child belongs. To help establish whether these adverse events are drug related, the same questionnaire will be administered once after each treatment round, to 286 children who are not part of the trial, recruited from nearby villages and matched for age to children in the trial.

### *3.9.2 Serious Adverse Events*

Any adverse experience occurring at any dose that results in any of the following outcomes:

- **Death**
- **Life threatening**- defined as an experience that places the patient or subject, in the view of the Investigator, at immediate risk of death from the reaction as it occurred. (Note: this does not include a reaction that, had it occurred in a more severe form, might have caused death).
- Requires inpatient **hospitalisation** or prolongation of existing hospitalisation.
- Results in a **congenital** anomaly or birth defect.
- Results in a **persistent or significant disability** or incapacity.
- **Important medical events** that may not result in death, be life-threatening or require hospitalisation may be considered as a serious adverse drug experience when, based upon appropriate medical judgement, they jeopardise the patient or subject and may require medical or surgical intervention to prevent one of the outcomes listed above (for example, an event may be defined as serious based on a deterioration in grade in Toxicity Tables).

Any serious adverse event that occurs during the study period will be reported to the Local Safety Monitor and Chairman of DSMB.

# *3.10 Morbidity surveillance during the rainy season*

Passive surveillance for malaria will be maintained throughout the transmission season. Parents/guardians will be encouraged to take their child to the health centre identified as being closest to their home at any time that their child becomes unwell. Project staff will be based at each of these health facilities to identify children in the trial and to ensure that they are seen, properly investigated and treated promptly. At each clinic visit, axillary temperature will be recorded using a digital thermometer and haemoglobin concentration measured using a Hemocue machine. A dipstick for diagnosis of malaria will be used if fever (axillary temperature of ≥ 37.5°C) or a history of fever within the previous 48 hours is present. In such cases, a thick blood smear will also be collected for subsequent confirmation of the diagnosis. Study subjects with documented fever (axillary temperature of ≥ 37.5°C) or history of recent fever and malaria parasitaemia will be treated with Coartem. The treatment of study subjects seen at the health centres for other conditions will be the carried out in accordance with national guidelines.

# *3.11 End of malaria transmission season cross-sectional survey*

Children enrolled in the study will be seen at the OPD clinic at the end of malaria transmission season for examination by a study physician and a finger-prick blood sample will be obtained for preparation of a thick blood smear and determination of haemoglobin concentration. A standardized questionnaire will be administered to the parents/guardians of the study subject, to collect information regarding illness that had occurred since the last visit, symptoms experienced, use of healthcare facilities and use of medicines. Information on the use of bed nets will be collected again at this visit.

*3.12 Study end-points:*

*3.12.1 Primary end points*

*Safety endpoint*

- Incidence of any adverse events during the observation period.

*Secondary end points*

- Mean Hb (g/dl) at the end of malaria transmission.
- Prevalence of malaria parasitaemia at the end of the malaria transmission season.
- Number of OPD attendances with malaria that meet the case definitions as indicated below during the surveillance period.

#### 3.13 Data management and analysis

All baseline, surveillance and laboratory data will be collected on forms designed for the trial. Field and laboratory staff will be trained to follow the procedures set out in a series of Standard Operating Procedures (SOPs). Key data will be double entered and inconsistencies checked; range checks will be used for standard variables. Primary analyses will be performed on an intention to treat (ITT) basis. Any child who receives a first dose of drug will be included in the ITT analysis. Children who are randomised but who do not receive any mediation will not be included in this analysis.

*3.14 Sample s-size calculations*

Drugs for IPT need to be acceptable and have low frequency of adverse events. If the frequency of adverse events is 20% in the SP/AQ group, a trial with 286 children per arm has 90% power to detect a halving in the frequency of adverse events, using a significance level of 0.05, or a reduction to 9% or less if a significance of 0.025 is used (to preserve an overall type 1 error rate of 5% for the primary endpoint when there are 2 comparisons, each alternative drug group with SP/AQ). If the frequency of adverse events is 15%, a reduction to 5% or less can be detected with the same power, and if the frequency is 10% a reduction to 2% or less can be detected. Based on experience from several studies the drop-out rate is not expected to exceed 15% and is likely to be less. Allowing for 15% drop out, a sample size of 286*3/(1-0.15)=1009 is needed.

# *3.15 Ethical considerations*

The findings from this study could influence the choice of drug combination for IPTc in areas of seasonal transmission. Study children will benefit from the intensive follow-up during the study period. In addition, all malaria cases detected will be treated with an effective antimalarial. This study will not interfere with or limit access of study participants to the malaria prevention programme provided by the department of state for health. All of the drug combinations to be used in the study, amodiaquine plus SP, piperaquine plus SP and ArtekinTM^m^ have been used extensively to treat or prevent malaria in young children and their safety profiles are well known.

*3.16 Oversight for the study*

A DSMB and Local safety monitor will provide oversight for the study

### Role of DSMB

The main role of the DSMB is to review and analyze clinical safety data collected during the trial and to assess unexpected or serious adverse events (SAEs).

The DSMC must be informed by the investigator of:

- serious and/or grade 3 adverse experiences occurring during the study (transmitted through the Local Safety Monitor)
- new information that may affect adversely the safety of the subjects or the conduct of the study.

###

### Role of Local Safety Monitor

The overall role of the Local Safety Monitor, who will be an experienced clinician, will be to support the clinical investigators and to act as a link between the investigators and the DSMB. All grade 3 adverse events probably or suspected to be related to trial medication and all serious adverse events will be reported to him in his capacity as a liaison to the DSMB. His involvement will be particularly important when decisions have to be made quickly.

**4.0 References**

Assessment of safety of artemisinin compounds in pregnancy. WHO TDR/RBM 2002.

Charles O. Obonyo, Elizabeth A. Juma, Bernhards R. Ogutu, et al. Amodiaquine combined with sulfadoxine/pyrimethamine versus artemisinin-based combinations for the treatment of uncomplicated falciparum malaria in Africa: a meta-analysis. Trans R Soc Trop Med Hyg, 2007, 101, 117—126.

Alonso, PL, Lindsay SW , Armstrong Schellenberg JR, et al. A malaria control trial using insecticide-treated bed nets and targeted chemoprophylaxis in a rural area of The Gambia, West Africa. The impact of the interventions on mortality and morbidity from malaria. Trans R Soc Trop Med Hyg 1993 87 Suppl 2: 37-44.

Badara Cissé, Cheikh Sokhna, Denis Boulanger, et al. Seasonal intermittent preventive treatment with artesunate and sulfadoxine-pyrimethamine for prevention of malaria in Senegalese children: a randomised, placebo-controlled, double-blind trial. Lancet. 2006; 367: 659–67

Brasseur, P, Agnamey P, Ekobo AS, et al. Sensitivity of Plasmodium falciparum to amodiaquine and chloroquine in central Africa: a comparative study in vivo and in vitro. Trans R Soc Trop Med Hyg, 1995. 89: 528-30.

Chandramohan D, Owusu-Agyei S, Carneiro I, et al. Cluster randomised trial of intermittent preventive treatment for malaria in infants in area of high, seasonal transmission in Ghana. BMJ. 2005 ;331: 727-33.

Chen, G, Absorption, distribution and excretion of ^14^C-piperaquine phosphate and ^14^C-piperaquine in human body Journal of Shangai, The Second Military Medical University, 1981: 47-48.

Dicko A, Sagara S, Sissokop MS, et al. Impact of intermittent preventive treatment with sulfadoxine pyrimethamine targeting the transmission season on the incidence of clinical malaria in children aged 6 months to 10 years in Kambila, Mali. Amer J Trop Med hyg 2004: 71: supplement.

Denis, M.B, Davis TM, Hewitt S, et al. Efficacy and safety of dihydroartemisinin-piperaquine (Artekin) in Cambodian children and adults with uncomplicated falciparum malaria. Clin Infect Dis, 2002. 35: 1469-76.

Eckstein-Ludwig, U, Webb RJ, Van Goethem ID, et al, Artemisinins target the SERCA of Plasmodium falciparum. Nature, 2003. 424: 957-61.

Greenwood, B. M. and H. Pickering. A malaria control trial using insecticide-treated bed nets and targeted chemoprophylaxis in a rural area of The Gambia, west Africa. 1. A review of the epidemiology and control of malaria in The Gambia, west Africa. Trans R Soc Trop Med Hyg 1993. 87 Suppl 2: 3-11.

Greenwood B. The use of antimalarial drugs to prevent malaria in population of malaria-endemic areas. Am J Trop Med Hyg 2004; 70: 1-7

Guo, X.B. Randomised comparison on the treatment of falciparum malaria with dihydroartemisinin and piperaquine ]. Zhonghua Yi Xue Za Zhi, 1993. 73 : p. 602-4, 638.

Hung TY, Davis TM, Ilett KF. Measurement of piperaquine in plasma by liquid chromatography with ultraviolet absorbance detection. J Chromatogr B Analyt Technol Biomed Life Sci. 2003. 5;791 :93-101.

Karema C, Fanello CI, van Overmeir C, et al. Safety and efficacy of dihydroartemisinin/piperaquine (Artekin) for the treatment of uncomplicated Plasmodium falciparum malaria in Rwandan children. Trans R Soc Trop Med Hyg. 2006 ;100:1105-11.

Laing AB. The impact of malaria chemoprophylaxis in Africa with special reference to Madagascar, Cameroon, and Senegal. Bull World Health Organ 1984; 62 (suppl): 41–48.

Lan CX, Lin X, Huang ZS, Chen YS, Guo RN. In vivo sensitivity of Plasmodium falciparum to piperaquine phosphate assayed in Linshui and Baisha counties, Hainan Province. Zhongguo Ji Sheng Chong Xue Yu Ji Sheng Chong Bing Za Zhi. 1989;7:163-5.

Looareesuwan S, Wilairatana P, Vanijanonta S, Pitisuttithum P, Viravan C, Kraisintu K. Treatment of acute, uncomplicated, falciparum malaria with oral dihydroartemisinin. Ann Trop Med Parasitol. 1996 ;90:21-28.

Leonardi E, Gilvary G, White NJ, Nosten F. Severe allergic reactions to oral artesunate: a report of two cases. Trans R Soc Trop Med Hyg. 2001 ;95:182-3.

McGready R, Cho T, Keo NK et al. Artemisinin antimalarials in pregnancy: a prospective treatment study of 539 episodes of multidrug-resistant Plasmodium falciparum. Clin Infect Dis. 2001 15;33:2009-16.

Massaga JJ, Kitua AY, Lemnge MM, et al., Effect of intermittent treatment with amodiaquine on anaemia and malarial fevers in infants in Tanzania: a randomised placebo-controlled trial. Lancet. 2003 ;361:1853-60.

Macete E, Aide P, Aponte JJ, et al. Intermittent preventive treatment for malaria control administered at the time of routine vaccinations in Mozambican infants: a randomized, placebo-controlled trial.

J Infect Dis. 2006 ;194:276-85

Olliaro P, Nevill C, LeBras J, et al. Systematic review of amodiaquine treatment in uncomplicated malaria. Lancet. 1996. 2;348:1196-201.

Phillips-Howard PA, W.L. Serious adverse drug reactions to pyrimethamine-sulphadoxine, pyrimethamine-dapsone and to amodiaquine in Britain. Journal of the Royal Society of Medicine, 1990. 83(2).

Pringle G and Avery-Jones S. Observations on the early course of untreated falciparum malaria in semi-immune African children following a short period of protection. Bull World Health Organ 1966; 34: 269–72.

Parise ME, Ayisi JG, Nahlen BL, et al. Efficacy of sulfadoxine-pyrimethamine for prevention of placental malaria in an area of Kenya with a high prevalence of malaria and human immunodeficiency virus infection. Am J Trop Med Hyg. 1998 ;59:813-22.

Price R, van Vugt M, Phaipun L, et al. Adverse effects in patients with acute falciparum malaria treated with artemisinin derivatives. Am J Trop Med Hyg. 1999;60:547-55.

Shen, N. Preclinical toxicology study of new antimalarial compound II: Piperaquine phosphate and its combination 'Malaria-preventing No 3'. Journal of Shangai The Second Military Medical University, 1981(3): p. 41-48.

Shulman, C. E., E. K. Dorman, Cutt F, et al. Intermittent sulphadoxine-pyrimethamine to prevent severe anaemia secondary to malaria in pregnancy: a randomised placebo-controlled trial. 1999. Lancet 353 : 632-6.

Schellenberg, D, C. Menendez, Kahigwa E, et al. Intermittent treatment for malaria and anaemia control at time of routine vaccinations in Tanzanian infants: a randomised, placebo- controlled trial. 2001. 357: 1471-7.

Sowunmi, A., Ayede AI, Falade AG, et al. Randomized comparison of chloroquine and amodiaquine in the treatment of acute, uncomplicated, Plasmodium falciparum malaria in children. Annals of Tropical Medicine & Parasitology, 2001. 95: p. 549-558.

Tran TH, Dolecek C, Pham PM, et al. Dihydroartemisinin-piperaquine against multidrug-resistant Plasmodium falciparum malaria in Vietnam: randomised clinical trial. Lancet. 2004. 3;363:18-22.

Wang, X., X. Li, and S. Wang, Study of mutagenesis of hydroxypiperaquine phosphate with sister chromosome exchange rate assay. Journal of Shangai The Second Military Medical University, 1981: p. 281-283.

Yin, M., Y. Chen, and W. Liu, Journal of Shangai. The Second Military Medical University, 1981: p. 277-279.

Yang HL, Yang PF, Liu DQ, et al. [Sensitivity in vitro of Plasmodium falciparum to chloroquine, pyronaridine, artesunate and piperaquine in south Yunnan] Zhongguo Ji Sheng Chong Xue Yu Ji Sheng Chong Bing Za Zhi. 1992;10:198-200.

WHO (2000). Experts Committee report on Malaria. Report. Geneva, WHO: 1-74.

**Subject Information sheet for cases**

**MRC Laboratories**

Study: A randomised trial to compare the safety, tolerability and efficacy of three potential drugs combinations for intermittent preventive treatment in children aged 1 to 5 years in an area of seasonal malaria transmission in Upper River Division, The Gambia

(Sponsored by MRC Laboratories and Gates Malaria Partnership, London)

## Subject/Patient Information Sheet

Seek consent from the parent/guardian of the child using the following explanation and in a language that he/she understands:

Malaria is an important cause of death and serious illness among Gambian children. However, the risk of malaria can be reduced by dugs and impregnated bed nets.

Recently, it has been shown that giving a full treatment dose of an anti-malarial drug at specific times, regardless of the presence or absence of malaria germs in the body can reduce the risk of malaria in infants and children. MRC and partners are carrying out a study to evaluate the safety and efficacy of three different combinations of drugs for preventive treatment that be can be used to protect infants and older children from malaria.

We want to find 1009 children aged 1 to 5 years to take part in this study. The study has been approved by the Joint Gambia Government/MRC Ethical Committee.

If you agree that your child can join the study, he/she will be randomly assigned to receive one of three different types of antimalarial drug combinations monthly during September, October and November. The three drug combinations are amodiaquine plus SP, piperaquine plus SP or Artekin ^TM^ (dihdroartemisinin plus piperaquine).

All the three drug combinations are known to work well and have been used to treat patients with malaria in Africa and Asia without causing any major bad effects. So your child will receive good treatment whichever drug combination your child receives. However, all drugs have side effects. The unwanted effects reported with these drugs have been mild and not serious. The duration of the trial is about 6 months.

After each round of drug administration, you will be asked to wait with your child for about 30 minutes so that we can check your child has not vomited the medication before being taken home. A MRC field worker will visit your child three and seven days after drug administration to check that he/she remains well and to complete a side effect questionnaire.

At the time of the first dose in September and at the end of the study in December we will ask your child for finger-prick blood samples. This test will not do him/her any harm but may cause some pain at the site where blood is taken. In addition, we will take a finger-prick sample from him/her whenever he/she develops fever and /or symptoms compatible with malaria.

If your child is sick at any time during the trial, he/she will receive free treatment at the health centre. Any costs you might have had to pay for transportation to the health centre will be reimbursed to you.

Your child need only join this trial if you want to. You can ask as many questions as you like and your child can leave the trial at any time without you giving any reasons.

If the study doctor thinks that your child should no longer take part in the trial for health or other reasons, he can end his/her participation.

**CONSENT FORM**

Study: A randomised trial to compare the safety, tolerability and efficacy of three potential drugs combinations for intermittent preventive treatment in children aged 1 to 5 years in an area of seasonal malaria transmission in Upper River Division, The Gambia

Child’s name…………………………………… Subject study number ____________

The information sheet has been read to me/I have read and understood the information sheet and I have had a chance to ask questions about the study.

I understand that my child will be asked to take treatment for malaria once every month for 3 months.

I understand that if my child gets sick at any time during the trial, he/she can go to the local health centre to receive treatment from the health centre and MRC staff there. Information about the child will remain confidential and will be used only for the purposes of the trial.

I understand that my child will be asked to give finger-prick blood samples at the beginning and the end of the study and whenever he/she develops fever and /or symptoms compatible with malaria.

I understand that my child does not have to take part in the trial, and that he/she can leave the trial at any time, this would not affect the health care he/she or the immediate family receive while the trial is going on.

Signature or thumbprint of Parent or guardian of the

child… …………………………...

I have read the above to_____________________(name of parent or guardian of the

child) in a language he/she understands. I believe he/she has agreed that his/her child can take part in this study.

Signature of Field worker supervisor (or designate): _______________________

Date: __/____/____

Name in capitals: ________________________________

**Subject Information sheet for controls**

**MRC Laboratories**

Study: A randomised trial to compare the safety, tolerability and efficacy of three potential drugs combinations for intermittent preventive treatment in children aged 1 to 5 years in an area of seasonal malaria transmission in Upper River Division, The Gambia

(Sponsored by MRC Laboratories and Gates Malaria Partnership, London)

## Subject/Patient Information Sheet

Seek consent from the parent/guardian of the child using the following explanation and in a language that he/she understands:

Malaria is an important cause of death and serious illness among Gambian children. However, the risk of malaria can be reduced by dugs and impregnated bed nets.

Recently, it has been shown that giving a full treatment dose of an anti-malarial drug at specific times, regardless of the presence or absence of malaria germs in the body can reduce the risk of malaria in infants and children. MRC and partners are carrying out a study to evaluate the safety and efficacy of three different combinations of drugs for preventive treatment that be can be used to protect infants and older children from malaria.

The study has been approved by the Joint Gambia Government/MRC Ethical Committee.

1009 will be randomly assigned to receive one of three different types of antimalarial drug combinations monthly during September, October and November. The three drug combinations are amodiaquine plus SP, piperaquine plus SP or Artekin ^TM^ (dihdroartemisinin plus piperaquine).

After each round of drug administration, children enrolled in the study will be visited at home three and seven days after drug administration to check that they remain well and to complete a side effect questionnaire.

To help establish whether these side effects are related to treatment, we want to find 286 children who are not part of the treatment trial. These children will not receive treatment but will be visited at home three and seven days after each round of treatment to complete a side effect questionnaire.

If your child is sick at any time during the trial, he/she will receive free treatment at the health centre. Any costs you might have had to pay for transportation to the health centre will be reimbursed to you.

Your child need only join this trial if you want to. You can ask as many questions as you like and your child can leave the trial at any time without you giving any reasons.

If the study doctor thinks that your child should no longer take part in the trial for health or other reasons, he can end his/her participation.

**CONSENT FORM FOR CONTROLS**

Study: A randomised trial to compare the safety, tolerability and efficacy of three potential drugs combinations for intermittent preventive treatment in children aged 1 to 5 years in an area of seasonal malaria transmission in Upper River Division, The Gambia

Child’s name…………………………………… Subject study number ____________

The information sheet has been read to me/I have read and understood the information sheet and I have had a chance to ask questions about the study.

I understand that my child will be visited at home on two occasions during September, October and November to check that he/she remains well and to complete a side effect questionnaire.

I understand that if my child gets sick at any time during the trial, he/she can go to the local health centre to receive treatment from the health centre and MRC staff there. Information about the child will remain confidential and will be used only for the purposes of the trial.

I understand that my child does not have to take part in the trial, and that he/she can leave the trial at any time, this would not affect the health care he/she or the immediate family receive while the trial is going on.

Signature or thumbprint of Parent or guardian of the

child… …………………………...

I have read the above to_____________________(name of parent or guardian of the

child) in a language he/she understands. I believe he/she has agreed that his/her child can take part in this study.

Signature of Field worker supervisor (or designate): _______________________

Date: __/____/____

Name in capitals: ________________________________
